# A High Throughput Integrated Hyperspectral Imaging and 3D Measurement System

**DOI:** 10.3390/s18041068

**Published:** 2018-04-02

**Authors:** Huijie Zhao, Lunbao Xu, Shaoguang Shi, Hongzhi Jiang, Da Chen

**Affiliations:** School of Instrumentation Science and Opto-electronics Engineering, Beihang University, Beijing 100191, China; hjzhao@buaa.edu.cn (H.Z.); xulb_buaa@163.com (L.X.); shisg_buaa@163.com (S.S.); chenda17@buaa.edu.cn (D.C.)

**Keywords:** hyperspectral measurement, 3D structure measurement, grating dispersion, stereo vison, agriculture

## Abstract

Hyperspectral and three-dimensional measurements can obtain the intrinsic physicochemical properties and external geometrical characteristics of objects, respectively. The combination of these two kinds of data can provide new insights into objects, which has gained attention in the fields of agricultural management, plant phenotyping, cultural heritage conservation, and food production. Currently, a variety of sensors are integrated into a system to collect spectral and morphological information in agriculture. However, previous experiments were usually performed with several commercial devices on a single platform. Inadequate registration and synchronization among instruments often resulted in mismatch between spectral and 3D information of the same target. In addition, using slit-based spectrometers and point-based 3D sensors extends the working hours in farms due to the narrow field of view (FOV). Therefore, we propose a high throughput prototype that combines stereo vision and grating dispersion to simultaneously acquire hyperspectral and 3D information. Furthermore, fiber-reformatting imaging spectrometry (FRIS) is adopted to acquire the hyperspectral images. Test experiments are conducted for the verification of the system accuracy, and vegetation measurements are carried out to demonstrate its feasibility. The proposed system is an improvement in multiple data acquisition and has the potential to improve plant phenotyping.

## 1. Introduction

The constantly increasing global population presents a tremendous challenge for agricultural production [1]. Improving crop varieties and developing precision agriculture have become key steps to increasing yield [2,3], inseparably linked to the ability to assess the phenotype of plants [4]. Currently, the measurements of thousands of plants are laborious and time consuming, and obtaining sufficient phenotypic data on a single plot remains problematic [1]. Thus, there is an urgent need to develop high throughput systems that allow plot-level measurements within seconds [3]. However, high quality plant phenotypic data and uncontrollable environmental conditions are two major challenges for field-based strategies [5]. Phenotyping of plants in controlled environments is an effective way to conduct genotypes selection according to differing phenotypes under controlled stress conditions. In addition, high throughput phenotyping in greenhouse has the possibility to relieve the bottleneck in gene discovery and crop improvement [6]. Among diverse measurements, hyperspectral and 3D measurement are essential manners to obtain traits. The former reveals the biochemical properties of crops while the latter provides the morphological characteristics of crops [7]. The combination of the two technologies plays an important role in vegetation physiology [8], precision agriculture [9] and cultural heritage [10]. 

In the past decades, numerous optical sensors have been developed to obtain spectral and 3D information in greenhouse and fields. These sensors can be classified into passive and active types. Active sensors are typically equipped with energy source to obtain spectral or depth information by projecting the signal onto objects and measuring the responses. 

For spectral measurement, active sensors (Tec5 AgroSpec, Trimble GreenSeeker, etc.) and passive sensors (ASD FieldSpec3, Specim ImSpector, Cubert UHD185, etc.) are often used for spectra acquisition. Hyperspectral imaging (HSI) enables the collection of the three-dimensional datacube (x,y,λ) that includes two spatial and one spectral information [11]. Now, HSI is intensively investigated for the measurement of crop nitrogen content, biomass, yield and crop stress [12] and can be a powerful tool to obtain the temporal dynamics of plant growth in greenhouse [5]. At present, there are three main types of imaging spectrometers available [13]. Whiskbroom spectrometers, which use a linear detector, capture the full spectral data of a pixel at each time and thus scan in two spatial domains to fill out the datacube. Pushbroom spectrometers use a 2D detector to obtain the spectral information of one spatial domain (x or y) and scan across the other one [14]. Staring spectrometers can obtain the full image of a certain wavelength, which is defined by a filter, and scan along the spectral dimension to complete the datacube [15]. The above three methods need scanning to accomplish the datacube acquisition either in the spatial or spectral domain. Furthermore, snapshot imaging spectrometers can obtain the entire datacube without scanning. In addition to the increased robustness and compactness, snapshot imaging also has the advantage of light collection, which provides potential for larger datacubes [11]. 

For 3D measurement, active sensors based on time-of-flight (TOF) or laser triangulation and passive sensors based on stereo vision or structure from motion (SFM) are common ways to acquire depth information. A lot of sensor technologies such as depth camera [16], lidar [17], structured light approaches [18], ultrasonic transducer [19], stereo camera system [20] are used to obtain 3D structures of plants. Point-based sensors (lidar, ultrasonic transducer) employ a narrow FOV that usually results in the loss of the highest point of crops [21]. Lidar can obtain a dense point cloud by increasing the number of scanning lines, while the cost will increase too. Depth cameras such as RGB-cameras offer a low-cost way to acquire 3D information [18], but due to the poor performance on sunny days, a shaded environment is required [22]. Close-up laser triangulation can provide 3D data of high precision, but a measuring arm or an auxiliary motion mechanism is needed. Simultaneously, stereo vision or SFM can obtain the dense point cloud through image processing with lower cost, but the algorithm is complex and the accuracy is limited. Thus, the accuracy, time efficiency, application field and cost should be considered when choosing a 3D sensor.

Most integrated systems combine the above-mentioned techniques for 3D structure and spectra measurement. For integrated system design, there are three main types: point-, line- and image-based styles. Point-based systems can obtain a 3D point and a spectral curve each time, and acquire the full data by whiskbroom. Zhao et al. designed an integrated system for auto-registered hyperspectral and 3D measurement by using the principle of point laser triangulation and prism dispersion [7]. The laser beam and the slit of the spectrometer were placed in the same plane. The reflected light of the laser and the sunlight was imaged on the detector by the prism through the same optical path. Therefore, at each time, the spectrum and depth of the same target point was obtained simultaneously without registration.

Line-based systems usually combine the line laser and the slit-based spectrometer, thus the entire data can be obtained through pushbroom. Behmann et al. developed an integrated system to generate 3D plant model with hyperspectral texture by combining several push-broom cameras and laser scanners. The sensors were geometrically calibrated to make sure that all the data were related to the same coordinate. Thus, depth information could be projected to the spectral image coordinate and assigned to the single pixels [13]. A similar approach was applied to the freshness predictions of fish using a structured-light system and a hyperspectral camera on a conveyor belt [23]. Brusco et al. proposed a system for automatic construction of spectral 3D models of architecture [24] using a point-based range finder and a slit-based spectrometer. The range finder was equipped with a rotating mirror to cover a 2D area and placed on the top of the spectrometer, ensuring that the sweeping region of the range finder coincided with the scanning area of the spectrometer. Thus, the models can be generated after data fusion without calibration. 

Image-based systems can extract 3D information directly from spectral images through SFM, and these systems need camera calibration only without registration. Aasen et al. generated digital surface model (DSM) using a unmanned aerial vehicle (UAV) and a snapshot camera [25]. The parameters such as plant height, chlorophyll, LAI and biomass were retrieved from the DSM to conduct vegetation monitoring. Zia et al. carried out the 3D reconstruction from hyperspectral images that captured by an acousto-optical tunable filter (AOTF) from multiple viewpoints. 3D point sets from the perspective images at each wavelength were generated first and then combined into a single hyperspectral 3D model [26]. 

The point- and line-based integrated systems are of high precision and suitable for precise modeling at leaf or plant level. Image-based systems with conveyor belts are appropriate for automatic high throughput phenotyping in greenhouse [27,28]. Currently, a variety of sensors are integrated on a moving platform to conduct phenotyping [4,29,30,31,32], thus geometric calibration and data registration are inevitable. In general, spectral and geometric characteristics are not measured simultaneously [13], a high precision Global Positioning System (GPS) and Inertial Measurement Unit (IMU) is needed [33], thus time and space accuracy becoming a challenge. If the data set can be obtained from a single sensor simultaneously in time and space, the accuracy of hyperspectral and 3D model will increase a lot [7].

In this study, we mainly aim to develop an integrated prototype that combines stereo vision based on triangulation for depth information acquisition and snapshot imaging based on grating dispersion for spectral data acquisition. Given that the system obtains data frame by frame, it can be applied for the simultaneous acquisition of the high throughput 3D structures and hyperspectral information of plants.

## 2. Background and Prototype

### 2.1. Hyperspectral Measurement

#### 2.1.1. Principle of Concave Grating Spectrometer

**Figure 1:**
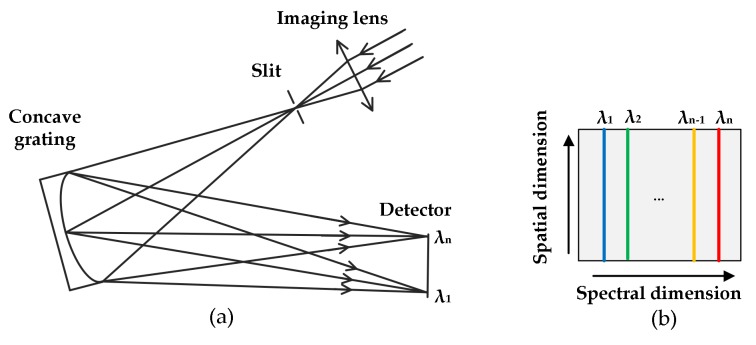
Schematic of a typical concave grating spectrometer. (**a**) Structure of the spectrometer. It consists of a fore lens, a slit, a concave grating and a detector. (**b**) Slit images on the focal plane. The horizontal axis represents the spectral dimension, whereas the vertical axis represents the spatial dimension.

Figure 1a illustrates the structure of the concave grating spectrometer. The incident light is imaged on the primary imaging plane by the fore lens, on which the slit lies as a field diaphragm. Then light coming out of the slit is dispersed by grating and focused on the detector. In contrast to plate grating, concave grating combines the functions of light dispersion and focusing, thereby ensuring that the spectrometer is compact and portable [34]. Moreover, the flat-filed design and aberration correction enable the planar detector to capture hyperspectral images. As shown in Figure 1b, a slit is imaged on the sensor with the spectral information horizontally dispersed and spatial information vertically spread.

#### 2.1.2. Snapshot Imaging

**Figure 2:**
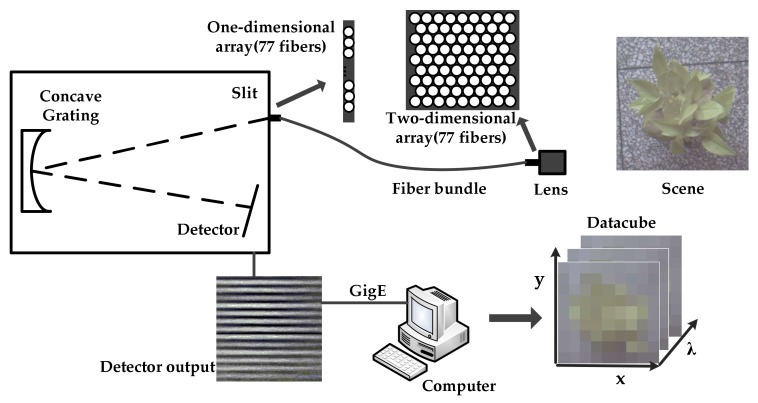
Schematic of the snapshot imaging system. A fiber array is used as the field diaphragm to transform a scene from two- to one-dimension.

Snapshot images can be obtained through several methods [11]. In particular, [35,36] proposed an appropriate approach called FRIS, in which a bundle of optical fibers was used for the transformation of a two-dimensional scene to one-dimensional strip that acted as the field diaphragm [37]. Figure 2 shows the schematic of the snapshot imaging system. It consists of the following components: an imaging lens, an optical fiber, one end of which is arranged in a square and the other end is arranged in a line, a flat-filed concave grating, and a monochrome detector. The squared end of the fiber is placed on the image plane of the lens, thus sampling a scene image at 77 positions. The other end that arranged in one-dimension is attached on the entrance plane of the spectrometer. Then, the incident light from the fibers is dispersed continuously along the spectral dimension, and separately along the spatial dimension. Thus, a series of stripes can be obtained from the detector, as shown in Figure 2. Each stripe contains the full spectral information corresponding to each sampling position of the scene. Therefore, a single-frame image can be reformatted into a datacube, of which each spectral image has 77 pixels. In this case, the resolution of the spectral image depends on the number of fibers. 

### 2.2. 3D Measurement

3D measurement based on typical binocular stereo vision consists of the following steps: camera calibration, stereo rectification, stereo matching, and 3D reconstruction [38]. Camera calibration aims at estimating the internal and external parameters of cameras. After stereo rectification, which reduces the 2D correspondence searing to 1D, homologous points in left and right images can be found through stereo matching, then 3D positions can be determined by triangulation using camera parameters [39].

### 2.2.1. Principle of Binocular Stereo Vision

**Figure 3:**
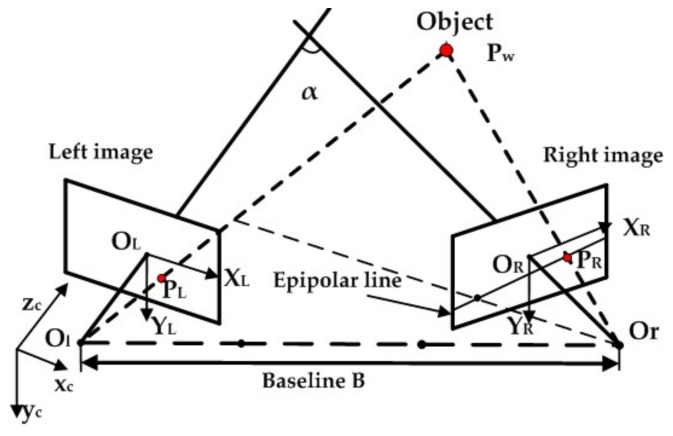
Principle of binocular stereo vision. $O_{l}{-x}_{c}y_{c}z_{c}$ and $O_{r}-\mathrm{xyz}$ represent the coordinates of the left and right cameras, respectively. $P_{w}{(x}_{c}{,y}_{c}{,z}_{c},1)$ denotes the homogeneous coordinate of object point in the left camera coordinate, $P_{L}{(X}_{L}{,Y}_{L},1)$ and $P_{R}{(X}_{R}{,Y}_{R},1)$ are the homogeneous coordinates of the projection points in the left and right image coordinates, respectively. B is the baseline of the stereo camera, α is the angle between two optic axes.

Binocular stereo vision can infer depth information with two cameras based on triangulation. Figure 3 illustrates the geometry of binocular stereo vision system. The object point $P_{w}\left(x_{c},y_{c},z_{c},1\right)$ is projected on two image planes at position $P_{L}\left(X_{L},Y_{L},1\right)$, $P_{R}\left(X_{R},Y_{R},1\right)$ through optical centers. That is, two half-lines defined by lens centers and projected points in two images intersect at one point in space. Their relationship can be described by the following equations:

(1) $$s_{l}P_{L}=A_{L}P_{w}$$

(2) $$s_{r}P_{R}=A_{R}[R|T]P_{w}$$

where R and T are the rotation matrix and translation vector between the left and right cameras, respectively, $A_{L}$ and $A_{R}$ are the intrinsic parameters of two cameras, $s_{l}$ and $s_{r}$ are the nonzero scale factors. When the parameters of the two cameras are known, which means that the spatial equations of the two half-lines are provided, the object point position under the left camera coordinate can be obtained. Figure 3 shows the common structure of binocular stereo vision system, which can be rectified into a standard model [40]. In this case, the two cameras are parallel. Therefore, the homologous points $P_{L}$ and ${\;P}_{R}$ are constrained on the same horizontal line of rectified images [41]. The coordinate of the object point is given by the following equations:

(3) $$x_{c}{=\mathrm{BX}}_{L}d^{-1}$$

(4) $$y_{c}{=\mathrm{BY}}_{L}d^{-1}$$

(5) $$z_{c}{=\mathrm{Bfd}}^{-1}$$

where B is the baseline, f is the focal length, and $d=X_{L}-X_{R}$ is the disparity.

### 2.2.2. Stereo Matching

Stereo matching is important to stereo vision, which uncovers pixel-wise correspondences between left and right images and subsequently generates the optimal map of disparities d(x,y) for all pixels (x,y) in the left image [42]. Furthermore, the search space is limited by the epipolar constraint. As shown in Figure 3, given a point ($P_{L}$) in the left image, the corresponding point in the right image lies along a line, particularly the epipolar line. Consequently, the constraint transfers the search space from the entire image into a line. After rectification, the homologous points can be found on the same horizontal lines through diverse matching algorithms. 

Currently, many matching algorithms can generate disparity maps, which consists of four steps, namely, cost computation, cost aggregation, disparity calculation, and refinement. The Semi Global Matching (SGM) [43] method is a widely used approach for the speed and dense points. In contrast to local method, SGM defines an energy function and optimizes it for the determination of the minimum cost paths by dynamic programming in some directions (from 4 to 16). The aggregated cost of every pixel can be gained by summing the costs of the minimum cost paths in all directions. The Semi Global Block Matching (SGBM) is an implementation of SGM provided by OpenCV and is based on matching blocks rather than pixels. In this study, SGBM is adopted for the generation of disparity maps. 

### 2.3. Prototype Design

**Figure 4:**
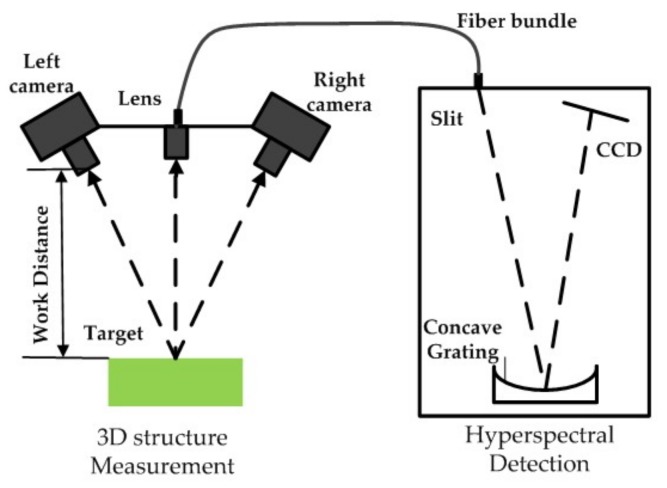
Schematic of the integrated system. It comprises two subsystems: a 3D system based on binocular stereo vision and a hyperspectral acquisition system using grating dispersion.

Figure 4 shows the structure of the integrated system. The left portion depicts the 3D structure measurement scheme, which consists of two cameras. The right portion illustrates the spectral detection component, in which the lens of the spectrometer is placed in the middle of the stereo cameras. The optic axes of the three lenses intersect at a distance of 1.2 m, thereby ensuring that the images captured by the two parts are centrally overlapping. Furthermore, one part of the reflected light from the target is captured by the stereo camera, from which a 3D point cloud is generated. Meanwhile, the other section is transmitted by a fiber bundle then dispersed by concave grating on the detector, from which hyperspectra are obtained. 

**Figure 5:**
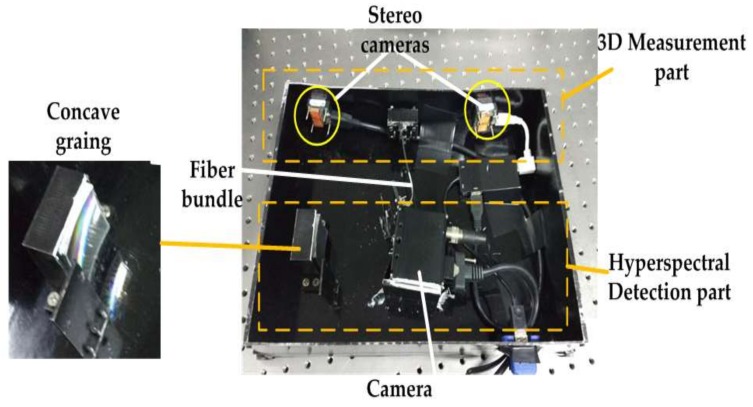
Photograph of the prototype.

Figure 5 shows a picture of the prototype. Its size and weight were 330 mm × 245 mm and 2.4 kg, respectively. The upper dashed box illustrates the 3D measurement component, which includes two Basler dart daA2500-14uc cameras, with 10° between two optic axes, and a baseline of 210 mm. The horizontal and vertical FOV of stereo cameras were 28°, and 8 mm lenses were used. The lower dashed box illustrates the hyperspectral detection element, in which a CMOS camera (HK-A5100-GM, Microview, Beijing, China) and grating were used. Furthermore, the numerical aperture (NA) of the fiber was 0.24, approximately 27.7° FOV, which ensured that the FOV was approximate to that of stereo cameras, and the fiber diameter was 125 μm. The software ran on a 3.2 GHz Core i5 PC without graphics processing unit (GPU) acceleration. Data acquisition of point cloud was performed at five frames per second. Moreover, stereo camera and spectral detectors captured the scene at the same time. An enlarged picture of the concave grating is shown on the left side of the figure.

### 2.4. Prototype Calibration

#### 2.4.1. Fiber Calibration

**Figure 6:**
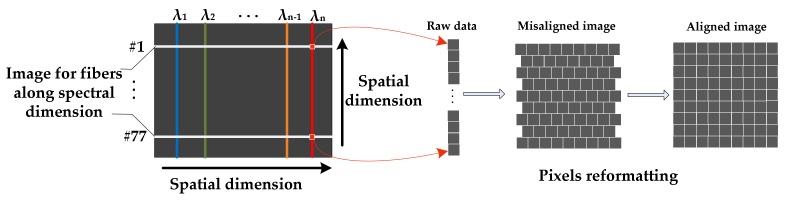
Schematic of a raw image of the proposed prototype.

As shown in Figure 6, the images of all fibers separately distribute along the spatial dimension due to the cladding and buffer that surround the fiber core, and continually distribute along the spectral dimension. In order to obtain the datacube, 77 digital numbers of each spectral band should be extracted from the raw image. Thus, the raw data can be rearranged into an image according to the original positions of the fibers. However, since the fibers were arranged in a staggered form, the pixels of the reformed image were misaligned. In order to generate an aligned image, bilinear interpolation was used. Hence, the aligned image had 9 × 9 pixels. During the process, the position of each fiber image in spatial dimension was recorded. The center of each strip was extracted through image processing, and digital numbers of each band λ can be calculated by averaging the values around the 77 centers with a certain window size (m×n), in which m depends on the width of each stripe and n depends on the width of each band. So, there is also a need to know the position and width of each spectral band. 

#### 2.4.2. Spectral Calibration

Spectral calibration focuses on determining the relationship between pixel position (N) and wavelength (λ). However, the sensitivity of fiber has an influence on spectral data. To generate an image, [36] used spectral sensitivity function and sensitivity ratio of each fiber to perform the correction. Considering that reflectance is widely applied in agriculture, the system can directly offer reflectance and reduce the influence of fiber. 

**Figure 7:**
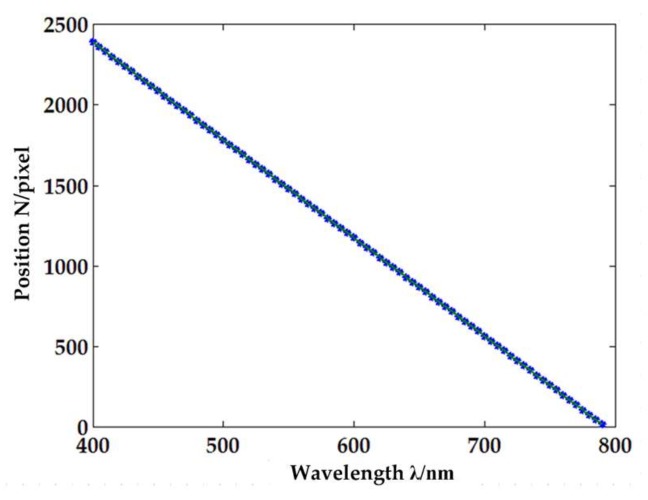
Relationship between pixel position N and wavelength λ for fiber #38.

A monochromator (SP2500, Princeton Instruments, Trenton, NJ, USA) equipped with a tungsten-halogen lamp acted as the standard light source. The mechanical range was 0–1400 nm with 0.2 nm accuracy and 0.05 nm repeatability. During the process, the drive step size was set to 5 nm, and two items were recorded at each step [44]: first, the pixel position (N) that corresponds to the peak of each band and current wavelength (λ); second, the full width at half maximum. After the calibration, the quadratic functions and spectrum resolution for 77 fibers were obtained. Figure 7 shows the fitting result for fiber #38. The pixel position N is linear to wavelength λ due to the linear dispersion of the grating. Table 1 shows the spectral resolution for fiber #38. The spectral range of the prototype is 450–790 nm.

#### 2.4.3. Stereo Camera Calibration

**Figure 8:**
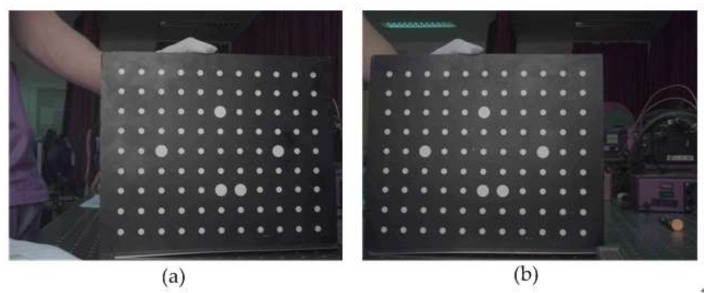
Calibration images of the left (**a**) and right (**b**) cameras.

**Figure 9:**
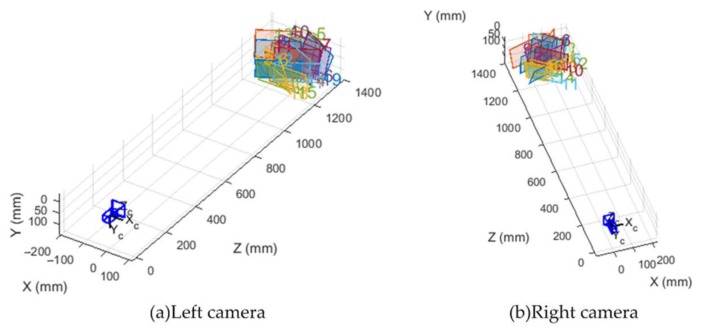
Relative positions of the calibration board to the left (**a**) and right (**b**) cameras. The optical center is the origin of the coordinate whereas rectangles in various colors represent the calibration boards in different positions.

In this step, we obtained the parameters of the cameras, particularly intrinsic and extrinsic parameters, and distortion coefficients, by using the method described by Zhang [45]. Figure 8 shows the calibration images of two cameras. The calibration board had a uniform distribution of 11 × 9 circular markers with known positions on the board. The calibration algorithm was based on the correspondence between the markers’ positions and their coordinates on the image plane. During the process, the calibration board was placed at various positions with diverse orientations. Finally, we calculated the re-projection errors to evaluate the accuracies of the calibration. The RMS values of re-projection error were 0.116 and 0.139 pixels for the left and right cameras, respectively. Figure 9 illustrates the calibration results. The figure depicts the relative positions, which are determined by the R and T parameters, between calibration boards and cameras. Intrinsic parameters are shown in Table 2, and extrinsic parameters are illustrated in Equation (6):

(6) $$\begin{array}{l} R=\left[\begin{array}{ccc} 0.9729103674 & 0.01359493293 & 0.2307825705\\ -0.01359465153\; & 0.9999063220 & -0.001591463205\\ -0.2307825871 & -0.001589057573 & 0.9730040454 \end{array}\right]\\ T={\left[\begin{array}{ccc} -215.9270416 & 2.937392166 & 27.7943499 \end{array}\right]}^{T} \end{array}$$

After calibration, the parameters of the prototype are listed as Table 3. In addition, the focal length and baseline of stereo camera can be calculated from the calibration results, which are 8.102 mm and 217.728 mm respectively. The prototype has 341 spectral bands in the range of 450 nm and 790 nm with 1 nm increment.

## 3. Experiment and Results

### 3.1. Accuracy Evaluation

To verify the accuracy of the data acquired by the prototype, a variety of test experiments were conducted. The wavelength accuracy of the prototype was evaluated by comparing the measurement data of a plant with a commercial spectrometer (FieldSpec3, ASD, Longmont, CO, USA), of which the spectral resolution was 3 nm @ 350–1000 nm and the FOV was 25°. Furthermore, the depth accuracy was evaluated by measuring the standard references.

#### 3.1.1. Wavelength Accuracy

**Figure 10:**
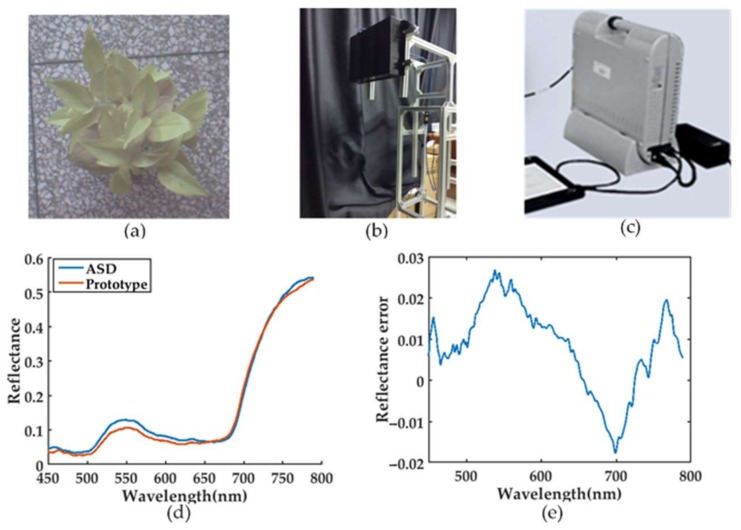
The spectral measurement with ASD and prototype. (**a**) *Epipremnum aureum*; (**b**) The prototype; (**c**) The ASD spectrometer; (**d**) Result comparison between prototype and ASD; (**e**) The errors relative to ASD.

An *Epipremnum aureum* plant acted as the object, as shown in Figure 10a. This experiment was carried out under laboratory conditions using a tungsten lamp. First, the spectral data of a standard diffusing reflector was acquired as the reference spectrum. Then, the average spectra of the plant were respectively obtained by the prototype and the ASD in the same position. Finally, the reflectance was calculated from the ratio between the spectra of the plant and that of the reference. Figure 10d shows the measurement results of prototype and the ASD. Since the prototype and the ASD had similar FOV and spectral resolution in the 450–790 nm range, the two measured spectra were almost overlapped. Furthermore, the root mean squared error (RMSE) was 1.34% in the range. 

#### 3.1.2. Depth Accuracy

**Figure 11:**
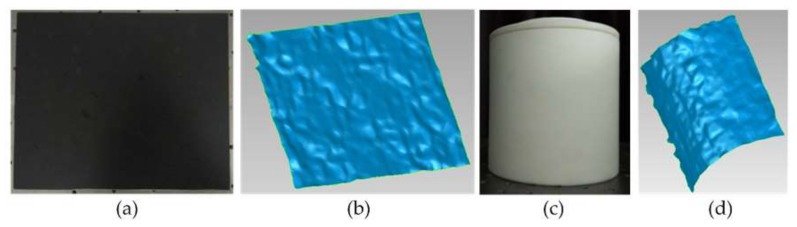
Standard plate (**a**) and column (**c**). The 3D point cloud of plate working surface (**b**) and column working surface (**d**).

The depth error caused by the disparity error [38] can be described as follows:

(7) $$\Delta z_{c}=\frac{-z_{c}^{2}\Delta d}{fB+z_{c}\Delta d}$$

where, $z_{c}$ is the working distance, *f* is the focal length, *B* is the baseline, and Δd is the disparity error. Thus, a wide baseline can improve depth accuracy whereas depth error increases with the measurement distance. The system errors contain calibration error (0.139 pixels) and matching error (no higher than one pixel). So, the maximum disparity error is 1.139 pixels. The pixel size in this study is 2.2 μm, then disparity error Δd can reach up to 2.506 μm. Furthermore, the focal length *f* is 8.102 mm, and the baseline *B* is 217.728 mm. If the working distance $z_{c}$ is 1.2 m, thus the error $\left|\Delta z_{c}\right|$ will be no higher than 2.042 mm according to Equation (7). In order to verify the accuracy, the standard plate and column were used for the evaluation of depth accuracy. However, the standard references lacked texture, thus, there was a need to provide features for the references to conduct the experiment [33]. The measurement was carried out by projecting a speckle image on their surfaces for texture generation. Figure 11 displays the targets and point cloud. 

**Figure 12:**
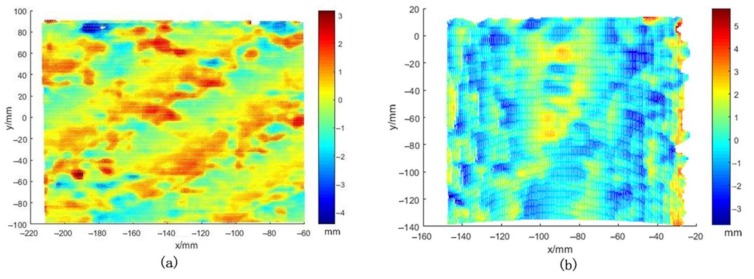
Evaluation of depth accuracy using the fitting method. (**a**) Error map of plate, ranging from −4.4 to 3.2 mm; (**b**) Error map of the column, ranging from −3.8 to 5.7 mm.

The surfaces of the objects are not smooth due to the measurement error. The errors of the measured plate were obtained by plane fitting, whereas the errors of the measured diameter were calculated by comparing the nominal value with that obtained by stereo vision. The RMSE at 1200 mm were 0.82 and 1.05 mm for the plate and column, respectively. In terms of three sigma standards, we use 3 times the RMSE to describe the accuracy, which was ±3.15 mm at 1.2 m. Figure 12 shows the fitting results of the plane and cylindrical surface. Obviously, errors between −3 and 3 mm account for a large proportion.

### 3.2. Vegetation Experiment

**Figure 13:**
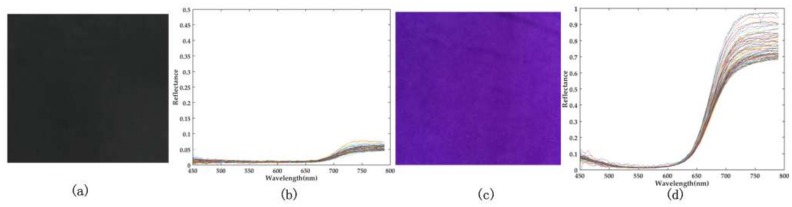
Black (**a**) and purple (**c**) backgrounds. The spectral data of the black background (**b**) and the purple background (**d**). The difference of reflectance between two backgrounds increases at 600 nm.

An *Epipremnum aureum* (plant 1) and a *Jasminum sambac* (plant 2) were used as experimental samples, and the experiment was conducted in laboratory condition. The 3D and spectral measurement of this system were both designed in a snapshot manner. Thus, the 3D structure and spectral image of the target were captured frame by frame. Given that the system focuses on acquiring information of the scene at the plant scale, the hybrid spectrum and structure above ground are presented. In the experiment, two kinds of vegetation in different backgrounds were measured. Figure 13a,b illustrate the black and purple backgrounds. Figure 13b,c show the spectral data for the two backgrounds respectively, and the purple background has a higher reflectance than that of the black one.

**Figure 14:**
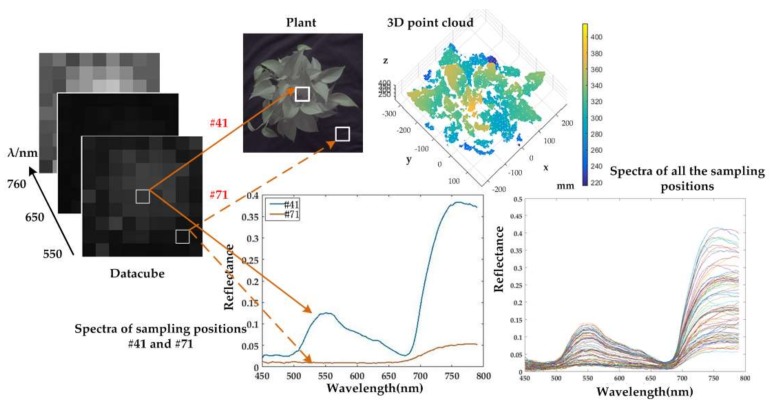
3D and spectral data of Plant 1. The top panel shows the image and 3D point cloud of the plant, whereas the bottom panel denotes the spectral curves. The left panel shows the datacube, of which the spectral images at the wavelength of 550, 650, and 760 nm are illustrated.

In Figure 14, the simultaneously obtained spectral datacube and 3D point cloud of plant 1 are illustrated. The scene image of the plant is sampled into 81 parts corresponding to the spectral image. The two patches that represent position #41 and #71 are taken as examples. 

**Figure 15:**
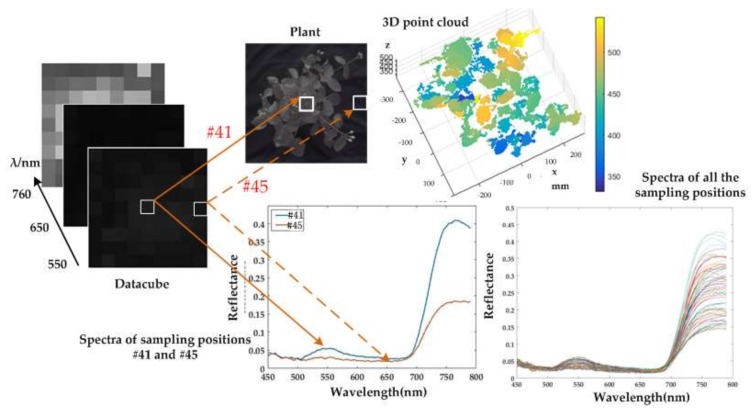
3D and spectral data of Plant 2. The top panel shows the image and 3D point cloud of the plant, whereas the bottom panel denotes the spectral curves. The left panel shows the datacube.

Position #41 represents a piece of plant area, which corresponds to a pixel in the spectral image. The data along the spectral dimension in that position can be illustrated as a spectral curve. Position #71 represents a part of the background, and the spectrum from that part is obviously different from that of plant. Furthermore, spectra of all the sampling positions are also illustrated in the figure. Thus, the high spatial resolution RGB image is transferred into a low spatial resolution hyperspectral image with 9 × 9 pixels. During the process, the prototype was placed directly above the sample. Given that the point cloud was extracted from two cameras at various positions, occlusions and discontinuities may have caused 3D data loss. It can be seen from the spectral curves that a reflection peak at approximately 550 nm and a reflection trough between 600 nm and 700 nm. In addition, the reflection increases sharply from 700 nm to 750 nm. Hence, the spectral images at 550, 650 and 760 nm are illustrated as examples. Figure 15 illustrates the experimental result of Plant 2. The reflection peak at approximately 550 nm is relatively low, while the reflection increases and differs from the background.

**Figure 16:**
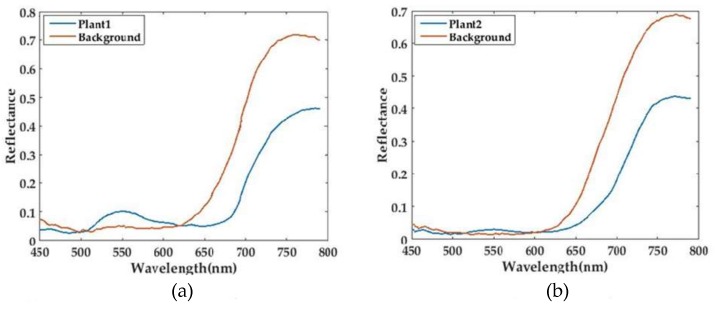

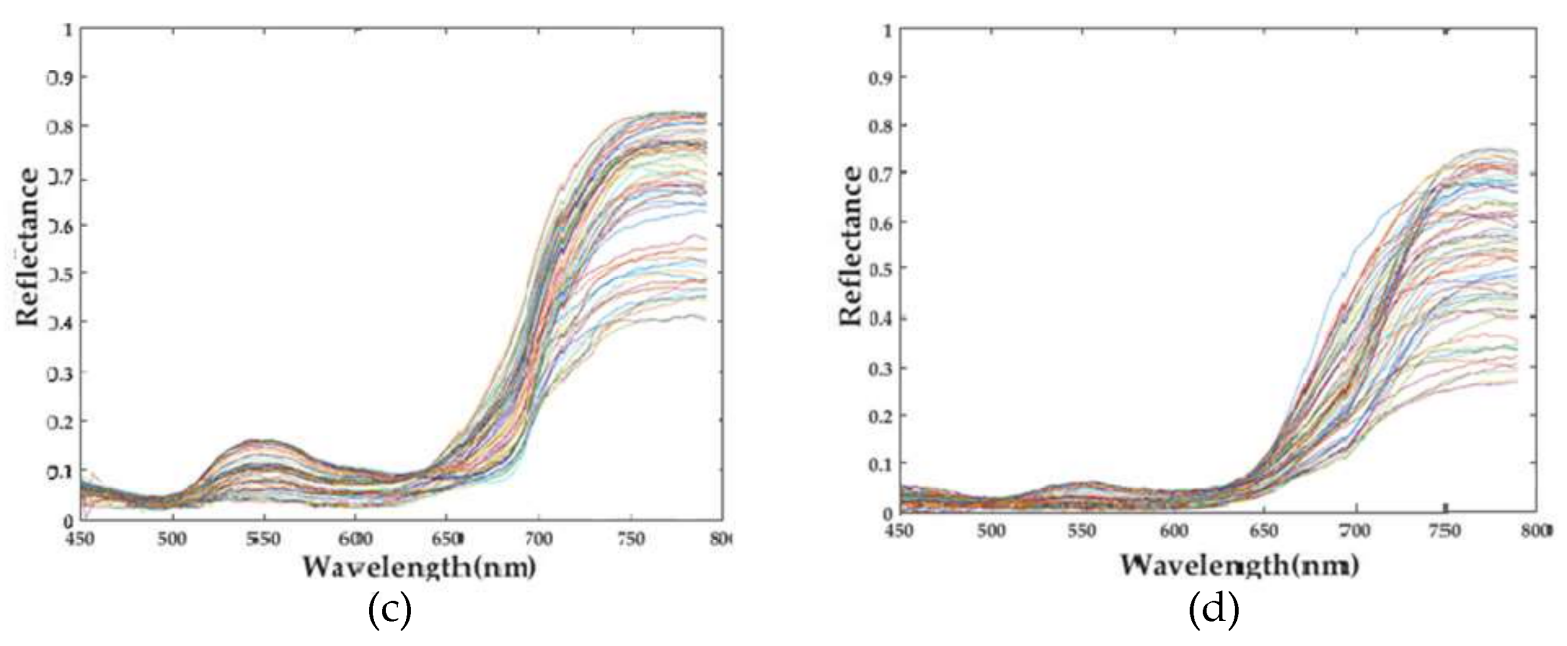
Spectral data of two plants with purple background. The upper sub-figures (**a**,**b**) show the spectra of two different sampling positions that correspond to the target and background respectively, while the lower sub-figures (**c**,**d**) display the spectra of all sampling positions.

Figure 16 shows the result of two plants with purple background. It can be seen from the figures that the reflectance of plants between 700 nm and 800 nm is relatively low (below 0.4) in Figure 14 and Figure 15, while, since some fibers receive the reflected light from both plant and purple background, the reflectance within that range increases in Figure 16 (some above 0.4). Furthermore, if a position only receives reflected light from plant, the reflectance will be relatively low. Hence, in Figure 16, there is a big range of spectra in all positions.

## 4. Discussion

### 4.1. Application Prospects

By comparing different approaches to achieve hyperspectral and 3D measurement, we combine stereo vision and spectral snapshot imaging to design an integrated system. Spectrometers, such as ASD that provides a mixed spectrum with 25° FOV, are widely used for reflectance measurement. However, they lack spatial resolution. On the other hand, the conventional approach used to obtain spectral image by scanning in the spectral or spatial dimension has issues in application. First, systems based on scanning along the spatial axis, particularly pushbroom devices, usually have slits, which limit imaging areas and scanning speeds. Second, although systems based on scanning along the wavelength axis, specifically the AOTF, are capable of acquiring spectra in a programmable manner, they are extremely expensive for widely application. Snapshot imaging can act as a compromise between these systems. This approach can obtain the entire spectral datacube each time. Thus, the time consumption of the measurement for plots will decrease.

Simultaneously, the prototype provides a high throughput way to acquire the dense 3D point cloud. The point-based 3D sensors need measuring arms or auxiliary motion mechanisms to perform the measurement, and the highest point may be lost due to the narrow FOV. Stereo vision, depth camera and SFM are suitable to acquire depth information in a high throughput manner. However, low-cost depth cameras perform poorly on sunny days, and SFM usually requires high precision GPS-IMU navigation. In addition, using different sensors to acquire multiple traits has some problems. First, different fields of view mean different measurement areas, thus, the sensor of broad FOV has to sacrifice speed to cooperate with the narrow one. Furthermore, the same target is measured repeatedly. Second, some active 3D sensors need to project the light onto objects, potentially interfering with the spectrometers. Finally, the asynchronization in time among sensors can bring errors to the spectral 3D models. When the sensors are mounted on a moving platform or the leaves are swaying, it is hard to acquire the combined data of the same target if the sensors are not measuring simultaneously. Therefore, the measurements of different sensors should be conducted with smallest delay and cooperate with each other. The development of integrated system will be of great help for multiple traits measurement and of great potential in agriculture. 

### 4.2. Limitations of This Study

The experiments were carried out to demonstrate the accuracy of the prototype and the feasibility of simultaneously capturing hyperspectral and 3D data. However, the prototype has several problems to be solved.

First, the frame rate is relative low. At present, the system can only work at five frames per second due to the high complex algorithm of stereo matching. Meanwhile, the experiments are performed on a PC without any acceleration. To realize real-time acquisition, the algorithm should be improved and implemented on the GPU. 

Second, the pixel number of the spectral image is small. The spectral images are obtained through FRIS, of which the resolution depends on the number of fibers, thus, it can be modified by increasing the number or decreasing the diameter of fibers. If the diameter of the fiber is constant, the size of the detector needs to be enlarged to accommodate the increase in the number of fibers. On the other hand, if the size of the detector is fixed, reducing the diameter can lead to a reduction of the power of the incident light. Since the power is shared both spatially and spectrally, for the acquisition of high spectral resolution, the spatial resolution of the hyperspectral image should be relatively low. However, techniques, such as compressive sensing [46] and image fusion [35], can help increase the resolution. Third, the 3D image and the spectral image are not co-registered. Currently, the prototype can only provide hyperspectral and 3D information covering the same area. Since the spectral image has a limited number of pixels, the 3D image has to be resampled at the expense of image resolution. Hence, it’s better to conduct the co-registration after increasing the resolution of spectral image. 

Fourth, fibers cannot fill a 2D region completely due to the inactive parts and round shapes, so, the spectral images are not continuous in space. This problem can be solved by coupling the fibers to an array of lenslets [37]. 

Finally, the RGB and spectral images do not completely coincide because the 3D structures and spectral data are captured with distinct lenses. In order to completely capture the same scene, a beam splitter should be used.

## 5. Conclusions

In this study, we propose a high throughput prototype capable of simultaneously acquiring hyperspectral images and 3D structures. The spectral range is 450–790 nm with the resolution 3.1 nm @ 600 nm, and the depth accuracy is ±3.15 mm at 1.2 m. The hyperspectral and 3D measurement are performed with grating dispersion principle and binocular stereo vision respectively. The spectral images are captured through FRIS using 77 fibers, thus, the pixel number is limited. Additionally, since the 3D point cloud is recovered from only two perspectives, some structures of plant are lost due to partial occlusion. In the future, algorithms for increasing spectral resolution and multi-view stereo system will be developed.

Combining different types of information can offer multiple traits and open up new possibilities in crop monitoring. Therefore, developing a combined system in terms of hardware and software is a novel trend, ensuring that data from each sensor of the same target are matched at the area or plant scale and even at point scale. Systems that can offer information in a timely manner, cover large areas, have sufficient spatial/spectral resolutions, carry multiple data, and have reasonable costs are urgently needed in agriculture [47]. Hence, the development of integrated system that adapts existing technologies in novel way will continue to improve crop varieties and agriculture management.

## Figures and Tables

**Table 1:** Spectral resolution for fiber #38.

Wavelength (nm)	Resolution (nm)
450	4.6
500	3.4
600	3.1
700	2.8
790	2.6

**Table 2:** Intrinsic parameters of the left and right cameras.

Physical Meaning	Parameter	Camera	Values
Focal length in x,y direction	$\left(f_{x},f_{y}\right)$	l	(3683.05, 3682.42)
		r	(3689.58, 3689.36)
Principle point coordinates	$\left(u_{0},v_{0}\right)$	l	(894,79, 934.69)
		r	(917.71, 902.49)
Radial distortion parameters	$\left(k_{1},k_{2}\right)$	l	(−0.0514, 0.1951)
		r	(−0.0734, 0.5032)
Tangential distortion parameters	$\left(p_{1},p_{2}\right)$	l	(−3.124 × 10^−4^, −3.105 × 10^−3^)
		r	(−5.721 × 10^−4^, 2.883 × 10^−4^)

**Table 3:** Parameters of the integrated system.

Spectral Range	Spectral Resolution	Fiber Number	Spectral Band
450–790 nm	4.6–2.8 nm @ 450–570 nm 2.8–2.6 nm @ 570–790 nm	77	341
FOV	Depth Accuracy	Measuring Speed	Working Distance
28°	±3.15 mm @ 1200 mm	5 frames/s	1000–1400 mm
